# Acceptance and Commitment Therapy for the Treatment of Music Performance Anxiety: A Pilot Study with Student Vocalists

**DOI:** 10.3389/fpsyg.2017.00986

**Published:** 2017-06-15

**Authors:** David G. Juncos, Glenn A. Heinrichs, Philip Towle, Kiera Duffy, Sebastian M. Grand, Matthew C. Morgan, Jonathan D. Smith, Evan Kalkus

**Affiliations:** ^1^Ivyland Counseling CenterWarminster, PA, United States; ^2^Performance Enhancement CoachingSan Francisco, CA, United States; ^3^Department of Music, University of Notre DameSouth Bend, IN, United States; ^4^Grand School of MusicNew Hope, PA, United States; ^5^Department of Psychology, La Salle UniversityPhiladelphia, PA, United States; ^6^Penndel Mental Health CenterPenndel, PA, United States; ^7^Clinical PsyD Program, Rutgers UniversityNew Brunswick, NJ, United States

**Keywords:** music performance anxiety, acceptance and commitment therapy, pilot study, psychological flexibility, hexaflex, vocalists

## Abstract

This study investigated the use of Acceptance and Commitment Therapy (ACT) as a treatment for music performance anxiety (MPA) in an uncontrolled pilot design. ACT is a newer, “third-wave” therapy that differs from previous MPA treatments, because its goal is not to reduce symptoms of MPA. Rather, ACT aims to enhance psychological flexibility in the presence of unwanted symptoms through the promotion of six core processes collectively known as the ACT “Hexaflex.” A small group of student vocalists (*N* = 7) from an elite choral college were recruited using objective criteria for evaluating MPA. Participants received 12 ACT sessions, and their baseline functioning served as a pre-treatment control. Treatment consisted of an orientation to ACT, identifying experientially avoidant behaviors, facilitation of Hexaflex processes, group performances in which valued behaviors were practiced in front of one another, meditations, homework, and completion of self-report measures before, during, and after treatment (at a 1- and 3-month follow-up). Improvements were observed in participants' cognitive defusion, acceptance of MPA symptoms, and psychological flexibility at post-treatment and follow-ups. Students also appeared to improve their performance quality and reduce their shame over having MPA. These results add to existing research suggesting ACT is a promising intervention for MPA, while also highlighting how vocal students may be less impaired by physical MPA symptoms.

## Introduction

### MPA prevalence among instrumentalists and vocalists

Music performance anxiety (MPA) is a potentially debilitating condition affecting professional and student musicians alike. Prevalence estimates for professionals and students are comparable, with ~15–25% of professional orchestral musicians (Fishbein et al., 1988; van Kemenade et al., 1995; James, 1997) and 21–23% of university students (Wesner et al., 1990; Schroeder and Liebelt, 1999) reporting severe symptoms of MPA. Given their younger age, university music students may experience more severe MPA than professional musicians (Steptoe and Fidler, 1987). Newer research suggests this could be due to undergraduate musicians' having less experience than professionals in performing music at a high-level (Papageorgi et al., 2011; Biasutti and Concina, 2014). In light of this, early intervention with undergraduate musicians is of critical importance.

One group of musicians under-represented in the MPA treatment literature is vocalists, particularly student vocalists. It is unknown if MPA prevalence in vocalists and instrumentalists is the same, though research indicates choral musicians suffer high levels of MPA (Ryan and Andrews, 2009). Further details about vocalists' experience of MPA and response to treatment are still unclear, because studies using vocalist samples do not investigate MPA treatments (Kokotsaki and Davidson, 2003; Kenny et al., 2004; Ryan and Andrews, 2009) or they use combined samples of vocalists and instrumentalists (Nagel et al., 1989; Esplen and Hodnett, 1999). Unlike instrumentalists, vocalists have a unique challenge for clinicians to address—they rely on both their vocal and breathing apparatuses when performing, and both work differently when anxious than when calm. So, learning to sing without being impaired by physical MPA symptoms is increasingly important.

### Available MPA treatments

A variety of treatment options exist for MPA. Most commonly, as many as 31% of professional musicians make use of pharmacological treatments such as betaadrenoceptor blocking medications, or “beta-blockers,” to reduce physiological sensations associated with MPA such as palpitations and hyperventilation, e.g., Propanolol, Nadolol (Kenny et al., 2012). Also common are psychotherapeutic treatments such as Cognitive Behavioral Therapy (CBT) and Psycho-dynamically oriented therapy. CBT is a popular therapy involving cognitive restructuring and behavioral exposure that has been shown to effectively treat mild to moderate MPA (Clark and Agras, 1991; Osborne et al., 2007; Braden et al., 2015). Psycho-dynamically oriented therapy has also shown promise in treating more severe forms of MPA (Kenny et al., 2014, 2016; Kenny and Holmes, 2015; Kenny, 2016).

Alternatively, there are numerous other therapies that fall into two basic categories: (1) treatments that promote relaxation and improved physical health, such as biofeedback (Thurber et al., 2010), hypnosis (Stanton, 1994), yoga (Khalsa et al., 2009), meditation (Lin et al., 2008), progressive muscle relaxation (Kim, 2008), and the Alexander Technique (Valentine et al., 1995), and (2) expressive arts therapies, such as music therapy (Montello et al., 1990) and guided imagery (Esplen and Hodnett, 1999). While promising, these alternative therapies lack strong empirical support, and many suffer from methodological limitations that weaken conclusions about each treatment's effectiveness, i.e., lack of treatment manuals, lack of objective MPA assessment, lack of a full battery of assessment measures, and few follow-up assessments (McGinnis and Milling, 2005). Therefore, leading MPA researchers argue CBT with exposure is considered best practice for MPA (Kenny, 2011).

While CBT has the most empirical support of all MPA treatments, one of its main treatment components has been questioned for its therapeutic benefit. Research examining the effectiveness of cognitive restructuring (CR) has suggested disengaging from one's thoughts while doing CR tasks may actually relate more to the improvements in therapy, rather than changing the content of one's thoughts (Teasdale et al., 2002). Furthermore, a treatment like CBT may not adequately address musicians' needs, because it leads to outcomes more commonly desired by patients in clinical settings (reduction of anxiety symptoms) than those of musicians with MPA (improved performance quality). Studies examining the effectiveness of CBT with exposure as a treatment for MPA have not found improvements in participants' performance quality, in spite of significant reductions in MPA symptoms (Osborne et al., 2007; Braden et al., 2015). Given these shortcomings and the aforementioned limitations of MPA treatment studies, the need for a new and practical MPA treatment is strong.

### Acceptance and commitment therapy for MPA

Acceptance and Commitment Therapy (ACT; Hayes et al., 1999, 2011) is a newer, “third-wave” therapy that has already shown to be a promising MPA treatment with a professional drummer (Juncos et al., 2014) and a university violinist (Juncos and Markman, 2015). Third-wave therapies, i.e., ACT, Dialectical Behavior Therapy or DBT, Metacognitive Therapy, Mindfulness-based Cognitive Therapy for Depression or MBCT, are contemporary CBT therapies that promote mindfulness and acceptance of one's emotional distress, rather than mastery or control of one's symptoms, a goal of conventional CBT (Hayes, 2004; Kahl et al., 2012). In fact, efforts to control one's unwanted symptoms are viewed as problematic in the ACT model. According to ACT, pathology occurs when one repeatedly engages in behavior aimed to reduce the frequency of unwanted internal experiences, such as thoughts, sensations, memories, or emotions. Such efforts are considered forms of “experiential avoidance,” which is a defining feature for all anxiety disorders (Eifert and Forsyth, 2005). An alternative to avoidance is an acceptance of our unwanted private experiences as they are (uncomfortable thoughts, emotions, sensations, memories, etc.) and not as our minds say they are (dangers to be avoided).

Unlike other MPA treatments, the goal of an ACT treatment is not to reduce one's symptoms of anxiety or distress. Paradoxically, symptom reduction can occur in an ACT treatment, but it is not an explicit goal (Bach and Moran, 2008). Rather, ACT aims to increase “psychological flexibility” in the presence of unwanted symptoms by promoting mindfulness and acceptance of those symptoms. Psychological flexibility refers to the ability to stay present with emotional distress while simultaneously engaging in and persisting with valued behavior (Hayes et al., 2011). Similar to physical flexibility, it is achieved by persisting with discomfort, rather than escaping from it, until one becomes increasingly tolerant of it so that valued behaviors are no longer avoided. In fact, avoidance of emotional discomfort through repeated attempts to reduce or eliminate it creates psychological rigidity and prevents corrective learning that emotional distress is not inherently dangerous, rather it is merely uncomfortable (Eifert and Forsyth, 2005).

### Psychological flexibility and the ACT “Hexaflex”

**Figure 1:**
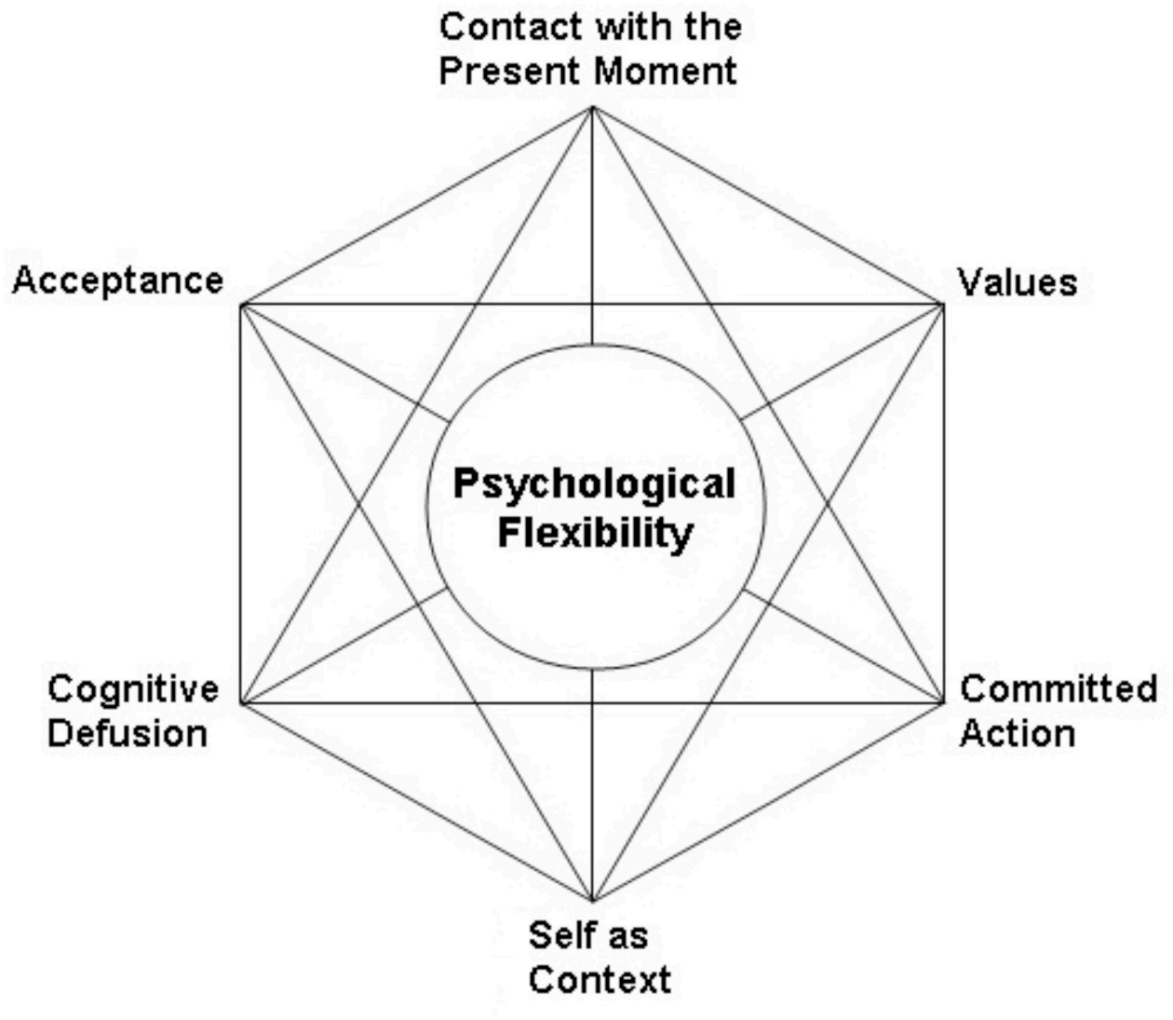
The ACT hexaflex.

Psychological flexibility is achieved through the combination of six processes targeted in an ACT treatment collectively known as the ACT “Hexaflex” (Figure 1). These include contact with the present moment (mindfulness), acceptance of one's experience, defusing from thoughts and feelings, adopting a contextual sense of self rather than a fixed self, identification of values, and committing to action consistent with one's values (Hayes et al., 2011). Mindfulness is a well-known construct that refers to the ability to remain aware of one's present-moment experience and accept it without judgment (Cardaciotto et al., 2008). It is promoted through regular meditation exercises both in-session and for homework. Acceptance refers to opening up to one's experience and observing it without reaction. It is promoted through experiential exercises like the Tug-of-War with the Anxiety Monster exercise (for this exercise, see Eifert and Forsyth, 2005).

Defusion is similar to mindfulness, because it involves the skill of observing one's thoughts, however it also aims to reduce their literality. According to the ACT model, our use of language can create suffering when we inadvertently believe our thoughts to be true. This possibility is referred to as the “language trap,” because our words can evoke the same emotional responses as their referents, through public or private (by our thoughts) language use (Eifert and Forsyth, 2005). Consequently, ACT clinicians strive to weaken the dominance of literal language through experiential exercises and metaphors (Hayes et al., 2011). For example, neutral words like “milk” are repeated numerous times until the client disengages from previous associations to the word. Then, emotionally charged words like “anxiety,” or “solo” are repeated until those words lose their literality. Defusion is also promoted by having clients verbalize their thoughts with the preface “I notice I'm having the thought that,” e.g., “I notice I'm having the thought that ‘I can’t breathe properly,”' or “I notice I'm having the thought that ‘I will fail my jury.”’

While a client is learning mindfulness and defusion skills, the clinician encourages him to adopt a sense of self as contextual rather than fixed. This Hexaflex principle is less concrete and is explained through the use of a chessboard metaphor (Eifert and Forsyth, 2005). The client is taught to identify with the board, or the space upon which the game occurs, instead of the pieces, or the content of the board. In this way, anxiety-related thoughts and feelings are experienced less personally, because they are viewed as passing experiences within one's self, rather than the actual self.

Once the previous Hexaflex processes have been promoted, the latter part of an ACT treatment focuses on identifying clients' values and increasing behavior in accordance with them. Values are defined as self-chosen directions one wants in life and can be identified through handouts and discussions. Values are distinguished from goals, which are concrete steps achieved along the way toward one's values (Eifert and Forsyth, 2005). The client is encouraged to engage in activities consistent with chosen values while simultaneously practicing the skills learned earlier in treatment. This portion of treatment involves repeated exposures to the emotional barriers that formerly prevented one from behaving in valued ways (Eifert and Forsyth, 2005). The client is encouraged to remain willing to experience those emotions in the service of chosen values, and efforts are made to eliminate experientially avoidant behaviors that prevent one from fully experiencing those emotional barriers.

### State of ACT research in treating similar disorders

Currently, ACT meets the “well-established” standard as a treatment for a variety of health problems including social anxiety disorder (Levin et al., 2017), a disorder very similar to MPA that is technically included in the same diagnostic profile (American Psychiatric Association, 2013). These standards were set forth by the American Psychological Association's (APA) Division 12 Task Force, in Clinical Psychology, in order to define empirically supported treatments within psychological research (see Chambless and Hollon, 1998). A well-established therapy must show either equivalency or superiority to another established treatment in at least two independently conducted and well-designed studies (Chambless and Hollon, 1998). Indeed, there have been two randomized controlled trials (RCT's) showing ACT is equally effective as CBT in treating social anxiety disorder (Kocovski et al., 2013; Craske et al., 2014). In both studies, ACT and CBT outperformed control, and there were no significant differences between treatments on self-reported symptoms of social anxiety (Kocovski et al., 2013; Craske et al., 2014). While CBT has more empirical support than ACT in treating social anxiety disorder, the existing ACT literature is certainly gaining ground. The authors of a recent meta-analysis of 39 RCT's involving ACT concluded it appears to be equally effective as CBT in treating anxiety disorders, as no significant differences were revealed between the two treatments (A-Tjak et al., 2015).

### Aim and hypotheses of the current study

This pilot study aimed to be the first to apply ACT to the treatment of MPA with a sample of university vocalists. In so doing, it aimed to elucidate how student vocalists may effectively sing without being impaired by MPA symptoms. It also aimed to investigate the effectiveness of ACT in reducing students' shame over having MPA, as many musicians with MPA commonly fear experiencing (and re-experiencing) shame while performing (Kenny, 2011). An uncontrolled pilot design was used, in which students' baseline functioning was compared to their functioning at post-treatment and two follow-ups. Similar to the design in the primary author's study involving a university violinist with MPA (Juncos and Markman, 2015), this study employed the following protocols: use of Eifert and Forsyth's (2005) treatment manual (*Acceptance and Commitment Therapy for Anxiety Disorders*), objective evaluation of MPA, pre- and post-treatment performances to assess for changes in performance quality, and a full-battery of self-report assessments.

Given the promising results of the primary author's two single-case studies (Juncos et al., 2014; Juncos and Markman, 2015), and given that ACT is now a well-established treatment for social anxiety disorder, there is reason to believe it may effectively treat the vocal students' MPA. Thus, the following three hypotheses were made: First, it was predicted there would be significant improvements in the measurable ACT hexaflex processes (mindfulness, defusion, acceptance, values identification, and committed action) at post-treatment compared to pre-treatment, and these improvements would be maintained at a 1- and 3-month follow-up. Second, it was predicted the ACT treatment would lead to significant improvements in performance quality at post-treatment, and thirdly, students' shame over having MPA would be significantly reduced at post-treatment, as indicated by reductions in state shame while performing in front of one another. Lastly, no predictions were made regarding MPA or related anxiety symptoms, given that symptom reduction is not a goal of ACT. However, anxiety measures were still administered to make comparisons to established norms for each measure.

## Materials and methods

### Participants

Ethical approval for the study was obtained from the Rider University Institutional Review Board. The participants were students (*N* = 7) from Westminster Choir College of Rider University, an elite music college dedicated to the study of vocal arts with one of the largest voice faculties in the world. There were two undergraduate students pursuing Bachelors degrees in Music with concentrations in voice studies and five graduate students pursuing Masters degrees with a dual emphasis in vocal performance and pedagogy. Six were female and one was male. Their ages ranged from 19- to 31-years-old (*M* = 23.29, *SD* = 3.73). Nine students enrolled originally, but two were unable to complete the full treatment due to scheduling conflicts. Vocal students at Westminster Choir College of Rider University are expected to perform in studio class every semester, in yearly choral hearings and juries, and in a recital at the culmination of their undergraduate or graduate career. Also, there are optional performances they may participate in yearly: operas, choir solos, auditions for the majority of Westminster Choir College's eight choirs, auditions for competitions and festivals, optional in-class performances, and recitals.

All students were eligible to participate because their self-reported MPA included each of several criteria common to anxiety disorders, i.e., cognitive symptoms, physiological arousal symptoms, behavioral avoidance, and distress/impairment from the anxiety problem (Brown and Barlow, 2002). Examples of cognitive MPA symptoms included worry about making mistakes during recitals or juries, worrying about becoming too tense to breathe properly, worry about becoming debilitated by anxiety and disappointing professors, and a narrowing of attention onto perceived threats, i.e., anxiety symptoms, mistakes, jurors' evaluations, etc. Examples of physiological arousal included shallow breathing, heart palpitations, tachycardia, dry mouth, increased physical tension, shaky hands and feet, and tightness in the chest. Examples of behavioral avoidance included overt behaviors (avoiding solo opportunities, avoiding optional performances in studio classes, avoiding auditions for desired roles, choosing a major with fewer performance requirements) and covert behaviors (avoiding eye contact with jurors during recitals, avoiding more challenging repertoire, avoiding expressing one's self and focusing only on technique, regularly holding onto the accompanist's piano while singing, regularly holding sheet music). All students reported their MPA was very distressing to them, and one student reported it also impaired her ability to pass a required piano course.

### Measures

#### ACT-based process measures

The Philadelphia Mindfulness Scale (PHLMS; Cardaciotto et al., 2008) was used to assess mindfulness. It is a 20-item measure comprised of two subscales: the Awareness subscale, which measures one's continuous monitoring of internal and external experiences, and the Acceptance subscale, which measures one's non-judgmental attitude toward one's experiences. It assesses the degree to which participants have experienced various statements about mindfulness in the past week, using a 5-point Likert scale (where 1 = never and 5 = very often). Higher subscale scores indicate higher levels of each construct. Internal consistency for the test is adequate for both the Awareness subscale (α = 0.75) and the Acceptance subscale (α = 0.82).

The Acceptance and Action Questionnaire-2 (AAQ-2; Bond et al., 2011) is a 10-item measure that was used to assess acceptance and action taken toward values (i.e., psychological flexibility). It asks participants to rate their level of agreement with various statements about those constructs, using a 7-point Likert scale (where 1 = never true and 7 = always true). Higher total scores indicate higher levels of psychological flexibility and acceptance. Furthermore, the AAQ-2 shows good internal consistency (α = 0.84).

The Believability in Anxious Feelings and Thoughts (BAFT; Herzberg et al., 2012) was used to assess cognitive defusion, and it requires participants to indicate on a 7-point Likert scale how much they agree with each of 30 statements related to fusion (where 1 = not at all believable and 7 = completely believable). Higher total scores on the BAFT reflect higher levels of fusion with one's anxious thoughts and feelings. It shows excellent internal consistency (α = 0.90).

The Valued Living Questionnaire (VLQ; Wilson et al., 2010) is a 20-item measure used to assess both the importance and consistency of one's personal values in a variety of domains. Items for the VLQ's Importance and Consistency subscales are rated on a 10-point Likert scale. First, participants indicate how important various values are to them, e.g., family, work, self-care, (where 1 = not at all important and 10 = very important). Next, they indicate how consistent their behavior was with each of those values in the past week (where 1 = not at all consistent and 10 = completely consistent). A VLQ Composite score is created by multiplying each value's Importance and Consistency scores and then averaging those cross-product scores. Higher Composite scores indicate higher congruence between one's values and behavior. The Importance and Consistency subscales show excellent (α = 0.93) and adequate (α = 0.60) internal consistency, respectively.

#### Symptom-based measures

The Anxiety Control Questionnaire (ACQ; Rapee et al., 1996) is a 30-item measure that assesses the degree to which participants agree with statements about their perceived control over internal anxiety-related emotions and external threats. It uses a 6-point Likert scale (where 0 = strongly disagree and 5 = strongly agree) and yields subscale scores for internal and external control and a total score for overall perceived control. Higher total scores indicate higher perceived control over both areas. Internal consistency for the total scale is good (α = 0.81).

The revised Kenny Music Performance Anxiety Inventory (KMPAI; Kenny, 2009) is a 40-item measure that assesses the degree to which participants agree with statements about anxiety-related discomfort associated with MPA. It uses a 7-point Likert scale (where 0 = strongly disagree and 6 = strongly agree, or vice versa, depending on the statement). Higher total scores indicate greater levels of anxiety and MPA-related distress. The revised KMPAI shows excellent internal consistency (α = 0.94). Its author also suggests a score of 105 or higher indicates clinically significant MPA (Ackermann et al., 2014).

The Experiential Shame Scale (ESS: Turner, 2014) is a 11-item self-report measure that assesses the degree to which one identifies with physical, emotional, and social components of the emotion of shame, using a 7-point Likert scale (where 1 = no presence of the symptom and 7 = extreme presence). Higher total scores indicate higher levels of state shame. The last question was modified to reflect one's willingness to discuss their music performance at the moment the ESS is taken. The ESS has adequate internal consistency (α = 0.72).

### Procedure

#### Intake procedures and consent

Interested students completed the standard intake procedures at Rider University's Counseling Center, on Westminster Choir College's campus. Intakes occurred midway through the Fall 2015 semester and included obtaining informed consent to be evaluated, a phone screening, and intake interview in which their psychological functioning was fully evaluated with the Structured Clinical Interview for DSM-V Axis I Disorders (SCID-5; First et al., 2015). Questions targeting their MPA symptoms (and addressing the aforementioned four categories of anxiety disorder symptoms) were designed by the principal author and added to the intake interview. Students were offered a spot in the study when they endorsed *all* categories of MPA symptoms, *and* when total scores on the KMPAI fell above the clinical cutoff, *and* when scores at on least one ACT-related measure were elevated by more than one standard deviation above the normative mean, *and* when MPA was the primary reason for their treatment. Student(s) whose clinical interviews revealed they also suffered with a more debilitating problem were excluded and referred for counseling instead, i.e., current depressive episode, substance abuse, eating disorder, etc. Students with comorbid anxiety disorders were not excluded, as MPA commonly co-occurs with other anxiety disorders and may even be part of those disorders, i.e., Social Phobia, Panic Disorder (Kenny, 2011). Eligible students provided informed consent to be enrolled and completed a second round of self-report measures at the end of the fall 2015 semester (in early December) to complete the baseline assessment.

#### Assessment schedule

Participants completed the full battery of assessment measures (AAQ-2, BAFT, PHLMS, VLQ, ACQ, KMPAI) at intake (baseline 1), at a second baseline point, at the first treatment session (pre-treatment), the fourth, eighth, and the twelfth session (post-treatment), and at a 1- and 3-month follow-up assessment. They completed them after each session and returned them within 24 h. Students were interviewed at both follow-up appointments to assess their overall impressions of the treatment.

#### Treatment

The 12-session ACT treatment coincided with students' Spring 2016 schedule, with the first session in late January and the last in late April. The treatment followed Eifert and Forsyth's (2005) manual as a guide, and it was modified wherever possible to apply to the treatment of MPA. The first half of treatment consisted of an orientation to the ACT model, creating an acceptance context for anxiety-related discomfort, and improving mindfulness and defusion skills, and identifying values and increasing values-consistent behavior. Regular meditations were used in and out of session to increase mindfulness and defusion skills. These skills helped to foster acceptance of MPA symptoms. The second half consisted of exposure exercises to put students in touch with feared thoughts and sensations, in-session performances that allowed students to practice valued behavior, and promotion of flexibility by increasing their willingness to experience MPA while simultaneously engaging in valued-behavior. After mindfulness and defusion skills were learned they engaged in “*FEEL* exercises” during therapy sessions (*F*eeling *E*xperiences *E*nriches *L*iving; Eifert and Forsyth, 2005). These were traditional exposure exercises in which the same physiological sensations that occurred during performances were deliberately created so they could practice accepting them with mindfulness and defusion skills. For example, students often experienced shortness of breath during performances. The principal author encouraged students to engage in an exercise designed to induce shortness of breath, i.e., breathing through a straw while walking up/down stairs, in order to practice mindfully observing the sensations while also defusing from thoughts associated with the sensations. Students were encouraged to defuse from anxious thoughts by verbalizing them with a specific preface, e.g., “I'm noticing I'm having the thought that ‘I am unable to breathe,”’ and “I'm noticing I'm having the thought that ‘I cannot perform this way.”’

In addition to traditional exposure exercises, the second half of treatment also included values-guided exposure exercises that enabled them to learn psychological flexibility. All students shared a value of being more expressive while performing, so the principal author developed a values-based technique with them to be used during performances, called the “Emotional Expression Technique.” This technique first had them mindfully rate the severity of their MPA as mild, moderate, or severe, using the Subjective Units of Distress rating scale (Hope et al., 2000). Then, depending on the severity, they would intentionally put either a mild or moderate amount of expressivity into their performance, using facial gestures and/or bodily movements of their choosing that were effectively timed and genre appropriate. The principal author defined “mild expressivity” as behavior noticeable to the performer only and “moderate expressivity” as behavior noticeable to both the performer and audience. Facial gestures and bodily movements were taken from a list of ways vocalists commonly express anxiety, compiled by the principal author and students. If students experienced severe anxiety or panic (which they did not), they were instructed to stop the performance and wait until calmer to resume.

The Emotional Expression Technique helped students choose when and how to engage in behavior consistent with their value of being expressive, even with MPA present. They practiced it while performing to the principal author during therapy sessions (immediately after *FEEL* exercises) and while performing in front of one another. They were encouraged to remain mindful and defuse from anxious thoughts and sensations related to using the Emotional Expression technique. ACT views these internal experiences as barriers to valued action, and psychological flexibility is learned whenever participants mindfully accept those barriers while re-directing energy into valued action.

Students completed homework exercises regularly, such as monitoring anxiety-related discomfort and willingness to experience discomfort, and identifying values with several handouts. Upon completion all students received a $25 gift card to an online retailer for participating.

#### Performance schedule

Students gave two video-recorded performances pre-treatment, in order to assess for baseline performance quality, one with accompaniment and one solo. Similarly, they gave two video-recorded performances at post-treatment. The principal author was the only audience member during the video-recorded performances. The accompanist was a graduate of the Royal Academy of Music in London with extensive experience providing accompaniment, teaching, and conducting. All students provided consent to be video-recorded and to be accompanied.

During the second half of treatment, students participated in weekly performances while continuing with individual sessions. They were given the option to perform either in front of therapists from Westminster Choir College's Counseling Center or their fellow participants in this study. All students chose to perform in front of their peers. Each peer performance lasted 1 h, and students were asked to perform a different piece each week, with the same accompanist from the video-recordings. Students had the opportunity to perform in six peer performances, but only four were required due to their busy schedules. Immediately after performing, each student completed an ESS form and remained in the audience for their peers.

#### Music performance quality

The Music Performance Quality Rating Form (MPQ; Educational Testing Service, 1998) was used to rate the quality of their pre- and post-treatment video-recorded performances. The MPQ uses a 5-item Likert scale to rate one's performance across six domains (pitch production, rhythm/tempo, technical skill, expressiveness, tone quality, and overall performance quality). There are five answer choices (1 = Awful, 2 = Could be better, 3 = Average, 4 = Very good, 5 = Excellent), and higher scores indicate higher performance quality. The MPQ gives two scores for each performance: the average is taken from the first five domains to produce a rating of *average* performance quality, and the sixth domain gives a rating of *overall* performance quality. No psychometric data was found, but it was used in other MPA treatment studies (Lin et al., 2008; Crawford, 2011).

Three independent raters rated the quality of the students' performances. Rater one was a professional cellist with extensive teaching and performing experience. Rater two was a professional performance coach with extensive experience with elite musicians and athletes. Rater three was a graduate of Westminster Choir College and a professional opera singer with extensive performance experience. All raters remained blind to the study's purpose and ordering of the performances. Students were instructed to perform four different pieces in their video-recordings, each 3–5 min in length and of equal difficulty level. Different pieces were used in order to reduce the potential bias that four recordings of the *same* piece may have on the ratings, i.e., students may have exhibited a practice effect if performing the same piece four times, which may have inadvertently revealed the ordering of the performances to raters.

#### Therapist description and adherence evaluation

The study's principal author served as the students' therapist. He was a post-doctoral resident with previous experience using ACT to treat musicians with MPA (see Juncos et al., 2014; Juncos and Markman, 2015), he attended three ACT training workshops prior to this study, and he received clinical supervision in ACT as a doctoral student. His adherence to Eifert and Forsyth's (2005) was rated by a clinical psychologist and a post-doctoral resident, both of whom have used ACT in their clinical practice. Each rater previously attended at least one ACT training, and they received further training here with a 30-min ACT training session. The raters used the Drexel University ACT/CBT Therapist Adherence and Competence Rating Scale, Revised Version (DUACRS-R). The DUACRS-R addresses ACT-specific therapist behaviors that may occur in session, and its ACT-specific subscale has good internal consistency (α = 0.86; McGrath, 2012).

## Results

### Baseline period

Scores on all self-report measures at both baselines were compared to scores at the first treatment session using a one-way ANOVA to assess for changes in functioning during the baseline period, or changes between the baseline period and first treatment session. No significant changes were found on any measure throughout this time period (all *p*'s > 0.05).

### Changes in psychological flexibility

Within-group changes on mean scores for ACT-based questionnaires (AAQ-II, BAFT, PHLMS, VLQ) were evaluated at post-treatment and follow-up assessments for significant improvements, using paired *t*-tests. Significant improvements were observed within students' pre-treatment mean scores (using both Baselines) and scores at post-treatment and follow-up assessments on the BAFT and AAQ-II, thereby supporting Hypothesis #1. See Table 1 for paired *t*-test results and *p*-values for scores on ACT-based measures. Effect sizes were large at post-treatment and at both follow-up points: Hedges' *g* for BAFT scores at post-treatment was 2.54 (using Baseline 1 data), *g* = 2.19 at 1-month follow-up, and *g* = 2.05 at 3-month follow-up, and Hedges' *g* for AAQ-II scores at post-treatment was 1.81, *g* = 1.48 at 1-month follow-up, and *g* = 1.34 at 3-month follow-up. Please note, one student did not return questionnaires at post-treatment and follow-up (student W2) therefore, that data was not included in *t*-tests at post-treatment and follow-up. However, the student completed the full 12-session treatment.

**Table 1:** Descriptive data and mean scores on ACT-based measures, as well as results of paired *t*-tests comparing pre-treatment means (Baseline 1 & 2) to means at post-treatment, 1-month follow-up, and 3-month follow-up.

**Schedule**	**Self-report measures**				
	**BAFT**	**PHLMS (Aware)**	**PHLMS (Accept)**	**AAQ-II**	**VLQ**
	*M* = 50.1^a^	*M* = 36.65^b^	*M* = 30.19^b^	*M* = 17.34^c^	*M* = 64.21^d^
	*SD* = 16.88	*SD* = 4.93	*SD* = 5.84	*SD* = 4.37	*SD* = 15.41
B1 (Pre-tx)	*M* = 80.57	*M* = 38.57	*M* = 26.43	*M* = 27.57	*M* = 51.56
B2 (Pre-tx)	*M* = 73.86	*M* = 39.57	*M* = 26.86	*M* = 25.86	*M* = 51.71
S12 (Post-tx)	*M* = 38.33	*M* = 38.17	*M* = 33.33	*M* = 17.67	*M* = 52.58
	*t*_(11)_ = 4.74^*^	*t*_(11)_ = 0.15	*t*_(11)_ = 1.76	*t*_(11)_ = 3.34^*^	*t*_(11)_ = 0.15
	*t*_(11)_ = 4.44^**^	*t*_(11)_ = 0.44	*t*_(11)_ = 1.84	*t*_(11)_ = 2.86^**^	*t*_(11)_ = 0.15
FU1	*M* = 40.5	*M* = 39.83	*M* = 33.83	*M* = 17.33	*M* = 56.62
	*t*_(11)_ = 3.99^*^	*t*_(11)_ = 0.46	*t*_(11)_ = 1.84	*t*_(11)_ = 2.66^*^	*t*_(11)_ = 0.68
	*t*_(11)_ = 3.61^**^	*t*_(11)_ = 0.08	*t*_(11)_ = 1.91	*t*_(11)_ = 2.26^**^	*t*_(11)_ = 0.75
FU2	*M* = 42.17	*M* = 38.83	*M* = 32.83	*M* = 19.17	*M* = 60.70
	*t*_(11)_ = 3.71^*^	*t*_(11)_ = 0.16	*t*_(11)_ = 1.57	*t*_(11)_ = 2.46^*^	*t*_(11)_ = 1.32
	*t*_(11)_ = 3.31^**^	*t*_(11)_ = 0.3	*t*_(11)_ = 1.61	*t*_(11)_ = 2.01	*t*_(11)_ = 1.52

Normative means and standard deviations for the

a *BAFT*,

b *PHLMS*,

c AAQ-II, and

d *VLQ were taken from non-clinical samples of undergraduates used in each measures' validation study*;

* *Statistically significant result at p < 0.05 level compared to B1 mean*;

** *Statistically significant result at p < 0.05 level compared to B2 mean; B, Baseline; S, Session; FU, Follow-Up*.

No significant differences were observed within pre- and post-treatment (and follow-up) scores on either PHLMS subscale or on the VLQ. However, there were clear improvements observed on students' PHLMS *Acceptance* subscale scores at post-treatment and at both follow-ups, though these changes were non-significant (see Table 1).

### Changes in music performance quality

**Figure 2:**
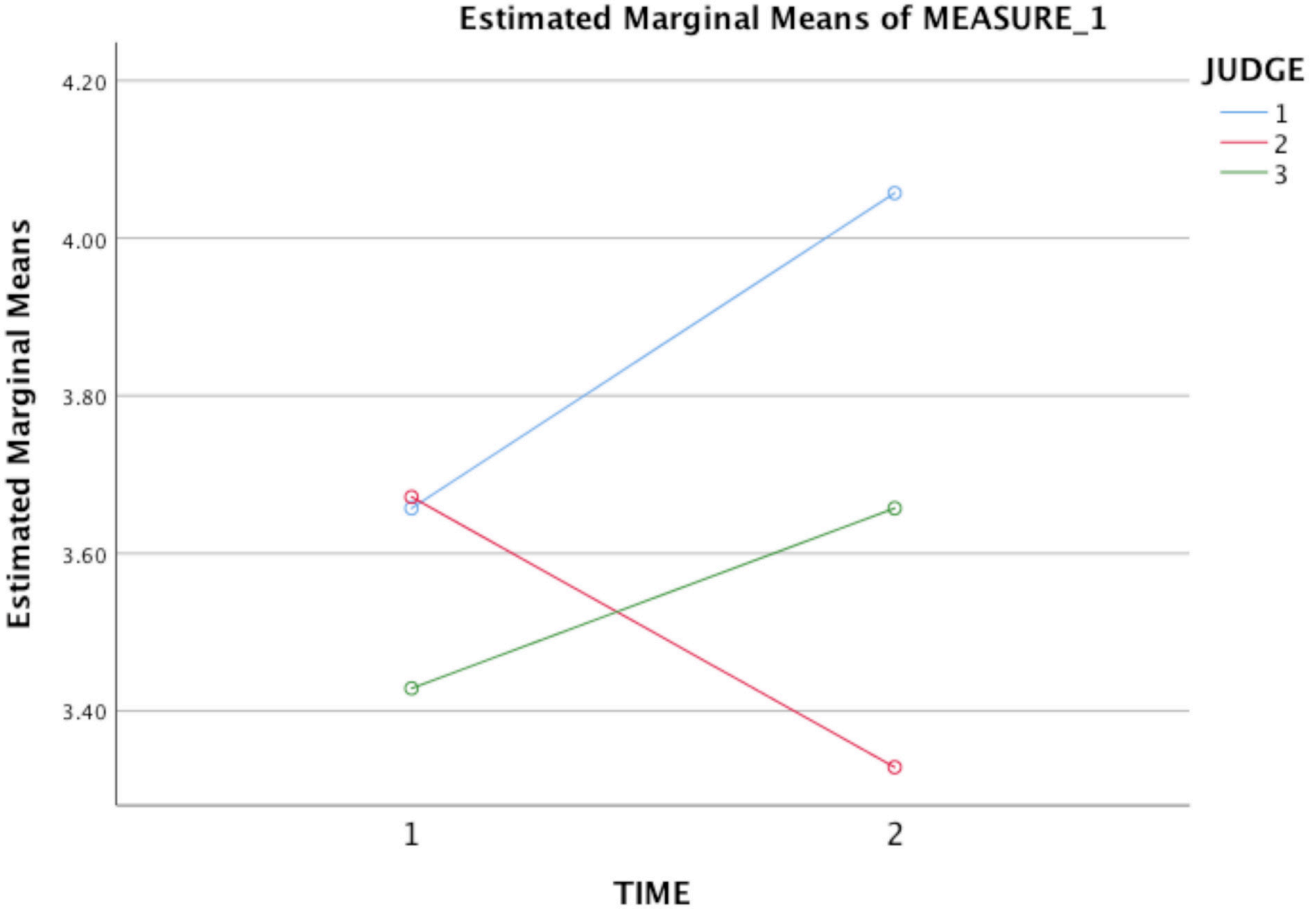
Mean performance quality ratings for students' pre- and post-treatment video-recordings using three independent raters' *average scores* on the MPQ. Time 1 = Pre-treatment; Time 2 = Post-treatment.

**Figure 3:**
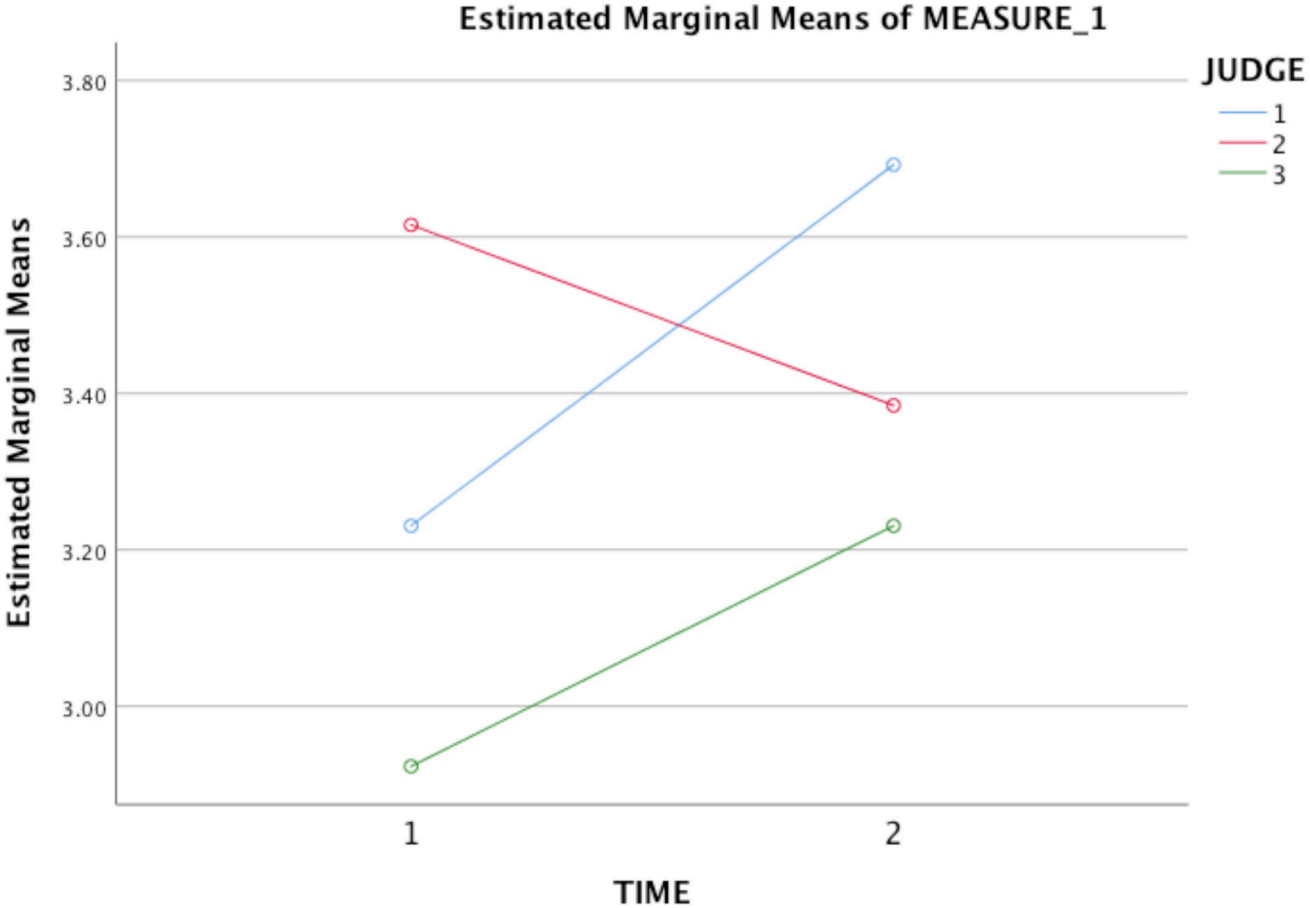
Mean performance quality ratings for students' pre- and post-treatment video-recordings using three independent raters' *overall scores* on the MPQ. Time 1 = Pre-treatment; Time 2 = Post-treatment.

The study's second hypothesis, that the ACT treatment would lead to significant improvements in students' overall performance quality, was supported by two of the three judges' ratings. Two repeated measures ANOVAs were conducted with performance time (pre vs. post) and raters (Judge 1, 2, or 3) as within-subject variables, and average MPQ ratings as the dependent variable in the first ANOVA and overall MPQ ratings in the second. Neither test produced a significant main effect of time: (1) *F*_(1, 6)_ = 0.67, *p* = 0.45 using average MPQ ratings, (2) *F*_(1, 6)_ = 1.05, *p* = 0.33 using overall MPQ ratings. However, when examining ratings at pre and post-treatment, Judges 1 and 3's ratings clearly indicated an improvement in performance quality had occurred with both types of ratings, whereas Judge 2's ratings showed the opposite trend. After removing Judge 2's ratings, a paired samples *t*-test was conducted using only Judge 1 and 3's MPQ ratings. The results were significant at *p* < 0.05 level for those judges' combined average MPQ ratings: *t*_(6)_ = 2.67, *p* = 0.037, with a large effect size (Hedges' *g* = 0.84). Results were significant at *p* < 0.10 level for those judges' combined overall MPQ ratings: *t*_(6)_ = 2.01, *p* = 0.09, also with a large effect size (Hedges' *g* = 0.90). See Figures 2, 3 for average and overall MPQ ratings, respectively. An intraclass coefficient value for Judges 1 and 3's overall MPQ ratings did not suggest adequate agreement between the two raters, ICC (3, 2) = 0.31.

### Changes in shame over having MPA

Within-group changes on mean scores for a questionnaire measuring state shame (ESS) were evaluated at students' first and last peer performances, using paired *t-*tests. Significant improvements were observed within students' ESS scores at the second assessment point, thereby supporting Hypothesis #3. ESS scores at each students' final peer performance (*M* = 3.47, *SD* = 0.25) were significantly lower than ESS scores at each students' first peer performance [*M* = 4.1, *SD* = 0.36; *t*_(11)_ = 3.89, *p* < 0.01]. A large effect size was found when comparing ESS scores at their first and last performances (*g* = 2.03).

### Changes in MPA and other symptoms

Within-group changes on mean scores for an MPA-based measure (KMPAI) and a related anxiety measure (ACQ) were evaluated at post-treatment and follow-up assessments, using paired *t*-tests. Significant reductions were observed within students' pre-treatment mean scores (using Baseline 1 & 2) and scores at post-treatment and follow-up assessments on both the KMPAI and ACQ. See Table 2 for paired *t*-test results and *p* values. Effect sizes were large at post-treatment and at both follow-up assessments: Hedges' *g* for KMPAI scores at post-treatment was 1.55, *g* = 1.61 at 1-month follow-up, and *g* = 2.19 at 3-month follow-up, and Hedges' *g* for ACQ scores at post-treatment was 1.64, *g* = 1.70 at 1-month follow-up, and *g* = 1.72 at 3-month follow-up.

**Table 2:** Descriptive data and mean scores on an MPA-based and other anxiety measure, as well as results of paired *t*-tests comparing pre-treatment means (Baseline 1 & 2) to means at post-treatment, 1-month follow-up, and 3-month follow-up.

**Schedule**	**KMPAI**	**Self-Report Measures ACQ**
	*M* = 93.5^a^	*M* = 96.1^c^
	*SD* = 39.1	*SD* = 18.9
	Cutoff = 105^b^	
B1 (Pre-tx)	*M* = 146.71	*M* = 74.86
B2 (Pre-tx)	*M* = 143.71	*M* = 80.29
S12 (Post-tx)	*M* = 115.17	*M* = 94
	*t*_(11)_ = 2.79^*^	*t*_(11)_ = 3.04^*^
	*t*_(11)_ = 2.46^**^	*t*_(11)_ = 1.88
FU1	*M* = 107.83	*M* = 95.33
	*t*_(11)_ = 2.89^*^	*t*_(11)_ = 3.12^*^
	*t*_(11)_ = 2.62^**^	*t*_(11)_ = 2.0
FU2	*M* = 101.33^***^	*M* = 95.67
	*t*_(11)_ = 3.94^*^	*t*_(11)_ = 3.15^*^
	*t*_(11)_ = 3.59^**^	*t*_(11)_ = 2.03

a *KMPAI normative data was from a group of professional orchestral musicians in Australia under age 30*;

b *Recommended cutoff score for determining clinically significant MPA*;

c *ACQ normative data was from non-clinical samples of undergraduates*;

* *Statistically significant result at p < 0.05 level compared to B1 mean*;

** *Statistically significant result at p < 0.05 level compared to B2 mean*;

*** *Below recommended cutoff score*.

### Individual responses to treatment

**Figure 4:**
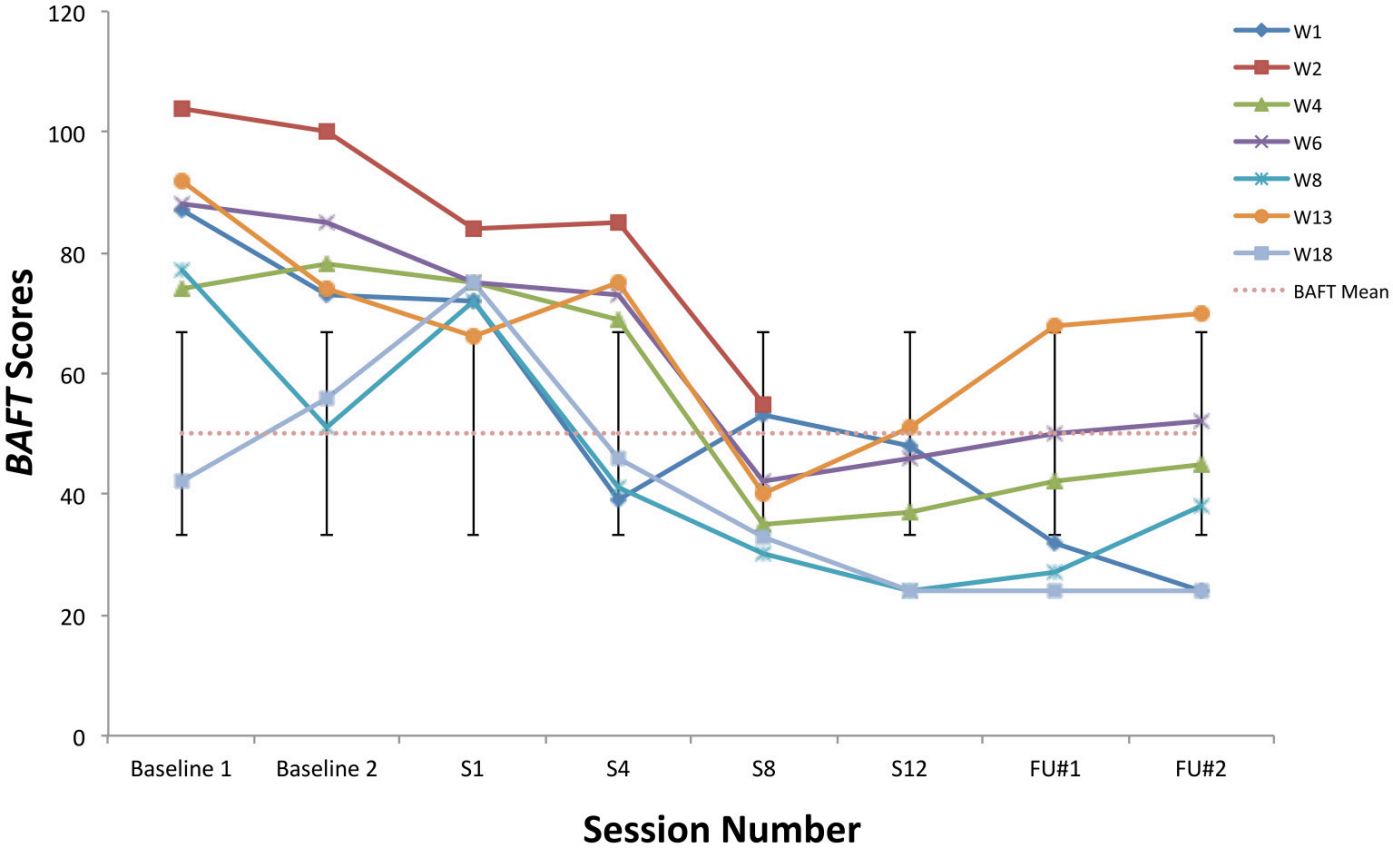
BAFT scores from the baseline period to post-treatment, 1- and 3-month follow-up points, including the non-clinical mean (50.10) and standard deviation bars (*SD* = 16.88; Herzberg et al., 2012).

**Figure 5:**
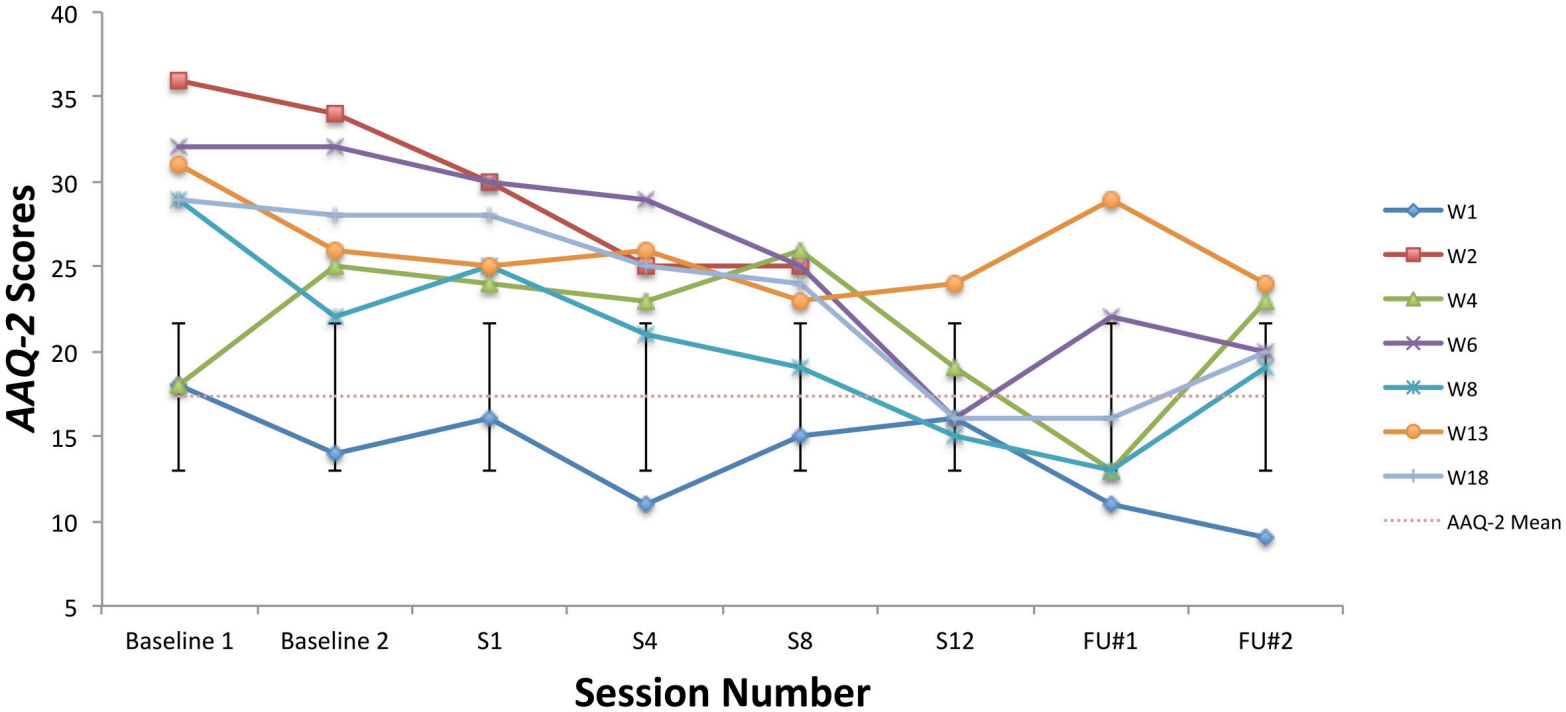
AAQ-II scores from the baseline period to post-treatment, 1- and 3-month follow-up points, including the non-clinical mean (17.34) and standard deviation bars (*SD* = 4.37; Bond et al., 2011).

**Figure 6:**
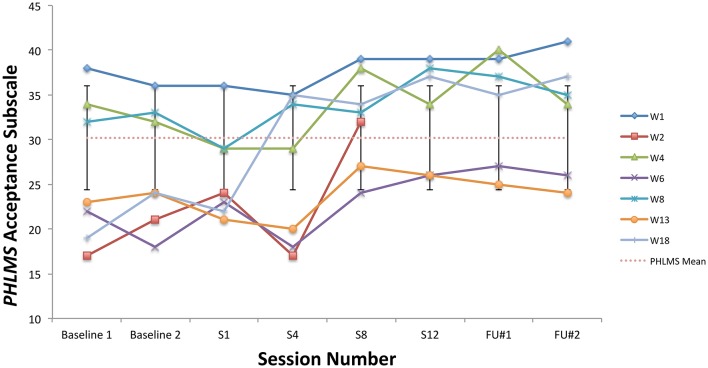
PHLMS *Acceptance* subscale scores from the baseline period to post-treatment, 1- and 3-month follow-up points, including the non-clinical mean (30.19) and standard deviation bars (*SD* = 5.84; Cardaciotto et al., 2008).

All students showed clinically significant improvements on at least one ACT measure at post-treatment, six students made significant improvements on at least two ACT measures (student W1 did not), and two improved significantly on three ACT measures (W6, W18). See Figures 4–**6** for scores on the BAFT, AAQ-II, and PHLMS *Acceptance* subscale, respectively. Here, a clinically significant improvement was identified when a student's score on a measure fell outside the normative range before treatment and inside that range after treatment, a metric commonly used in single-case designs (Kazdin, 2011).

Specifically, all seven students made clinically significant improvements on the BAFT between the baseline period and post-treatment (Figure 4). Four of seven students (W4, W6, W8, W18) made clinically significant improvements on the AAQ-II between the baseline period and post-treatment (Figure 5), and two of the other students made reliable improvements between the baseline period and sessions 8 and 12 (W2, W13). A reliable change index was calculated for these two students, and it confirmed improvements on the AAQ-II were not simply due to standard error of measurement, following criteria by Jacobson and Truax (1991). Also, scores for four of seven students (W2, W6, W13, W18) fell below the normative range on the PHLMS *Acceptance* subscale at the baseline period, and each advanced into the normative range at either session 8 or 12 (Figure 6).

**Figure 7:**
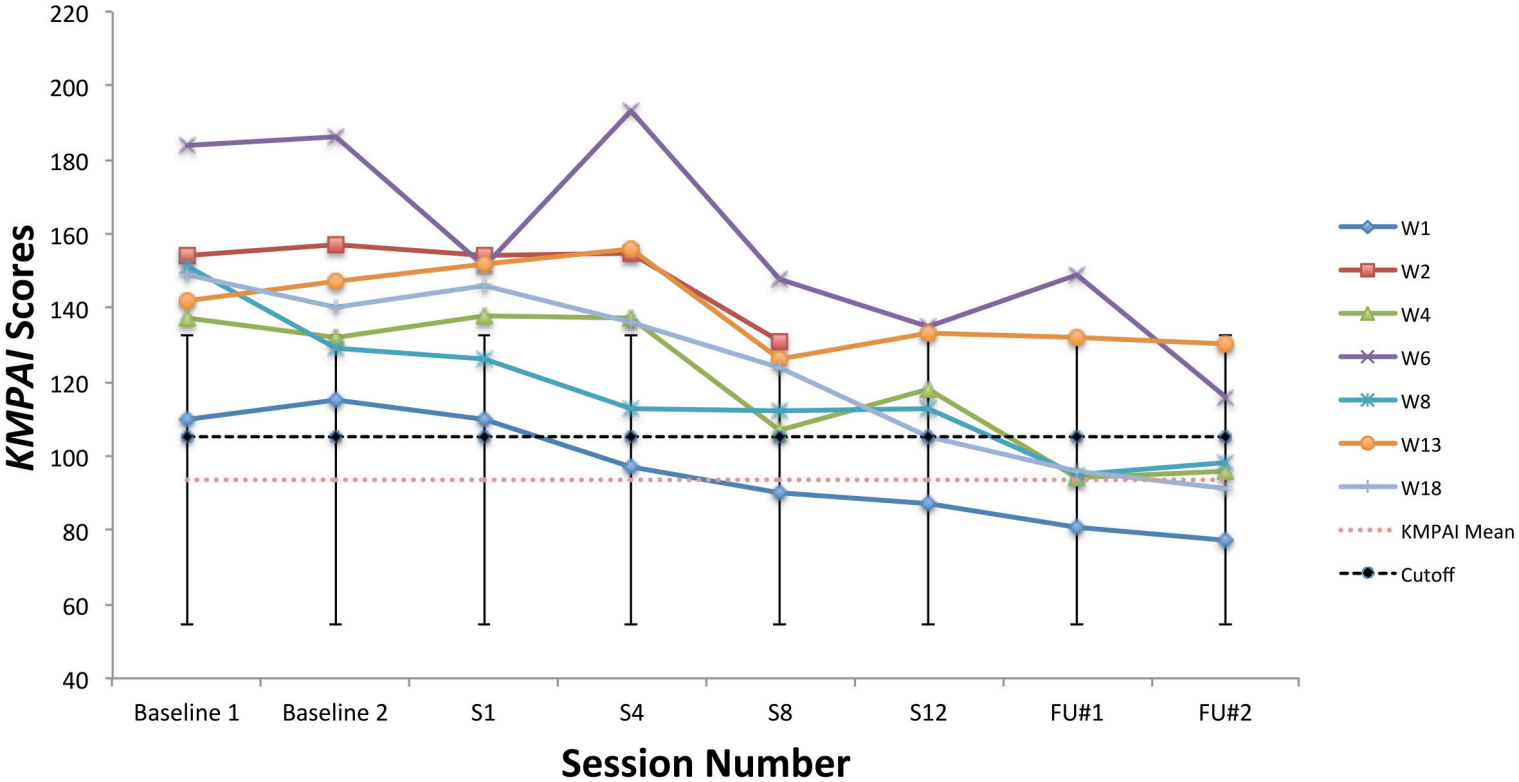
KMPAI scores from the baseline period to post-treatment, 1- and 3-month follow-up points, including the mean for musicians under age 30 (93.5), standard deviation bars (*SD* = 39.1; Kenny et al., 2012), and the recommended clinical cutoff score (105; Ackermann et al., 2014).

**Figure 8:**
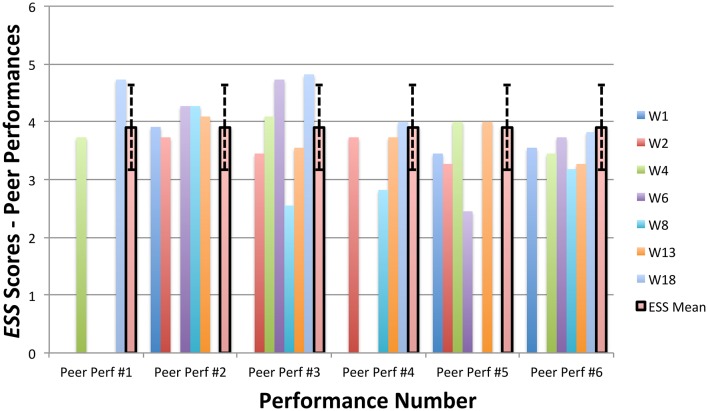
ESS scores from six peer performances with piano accompaniment, including the non-clinical mean (3.9) and standard deviation bars (*SD* = 0.73; Turner, 2014).

During the baseline period, all students' scores on the KMPAI fell above the recommended clinical cutoff score (105; Ackermann et al., 2014), whereas four of seven students' scores fell below the cutoff by the 3-month follow-up (W1, W4, W8, W18). See Figure 7 for KMPAI scores. Additionally, scores for six of seven students fell outside the KMPAI's normative range at the baseline period (all but W1), and each fell within the normative range at some point between session 8 and the 3-month follow-up point (Figure 7). W1 made a reliable improvement between the baseline period and the 3-month follow-up point. Scores for five of seven students fell below the ACQ's normative range during the baseline period (W1, W2, W4, W6, W13), and four of them made clinically significant improvements at post-treatment (W1, W4, W6, W13). Another student (W8) made a reliable improvement in ACQ scores from the baseline period to post-treatment. Two students' scores on the ESS fell outside the normative range during their first or second peer performance (W6, W18), whereas both students' scores fell within the normative range at their final performance (Figure 8). Another student made a reliable improvement between successive peer performances, i.e., W8 improved between the first and second performance.

When asked to share their impressions of the overall ACT treatment during the follow-up assessments, students reported an increased confidence in their performance skills, which they attributed to focusing more energy on valued action during performances and less energy on attempting to control MPA symptoms. They also reported the peer performances were very helpful, because they afforded them the opportunity to practice skills learned in therapy. Other noteworthy outcomes were the following reductions in experientially avoidant behaviors: all three students who were eligible to audition for next years' operas did so, two students began soloing more in their professional performances after treatment, one student deliberately avoided preparing for the peer performances so she could practice defusing from anxious thoughts about being unprepared, one student accepted an offer to perform in a master class at the famed Spoleto Music Festival, and another student deliberately watched her video-recorded performances after therapy (despite feeling anxious) because she wanted to practice mindfully accepting any shame-related thoughts that occurred. Lastly, students remarked the peer performances helped them to let go of their shame over having MPA, because they felt accepted by their peers.

### Therapist adherence and competence

Two raters were trained to use the DUACRS-R by the principal author. Once they achieved a high level of agreement (80%) with the principal author on an ACT training session, they evaluated eight sessions independently (chosen randomly) to assess the principal author's adherence to Eifert and Forsyth's (2005) manual. The intraclass coefficient value for their adherence ratings on the ACT-subscale did not suggest adequate consistency between raters, ICC (3, 2) = 0.31 or agreement, ICC (3, 2) = 0.25. However, the DUACRS-R measures adherence to the overall ACT model, rather than to Eifert and Forsyth's manual; it was chosen because it is the only known scale for measuring ACT-adherence. When asked to subjectively rate the principal author's adherence to manual, the raters reported he showed good adherence to the Eifert and Forsyth's recommended agendas for the eight sessions. Furthermore, they rated his overall competence as very good.

## Discussion

Consistent with the previous single-case designs in which ACT was used to treat MPA, this study's results indicated similar outcomes had occurred. First, students showed significant improvements in their ability to defuse from MPA-related thoughts at post-treatment and both follow-ups (see BAFT scores in Table 1), a result shared with the university violinist (Juncos and Markman, 2015) and the professional drummer (Juncos et al., 2014). Next, students showed significant improvements in their ability to accept MPA symptoms and take action toward values at post-treatment and both follow-ups (see AAQ-2 scores in Table 1), as did the violinist (Juncos and Markman, 2015). These processes, acceptance of unwanted experiences and defusion from them, are two proposed mechanisms by which ACT treatments increase psychological flexibility (Ciarrochi et al., 2010). Another proposed mechanism is a reduction in experiential avoidance. The improvements in defusion and acceptance, along with the noteworthy outcomes reported by students during the follow-up assessments, suggest they were engaging less in experientially avoidant behavior at post-treatment and both follow-ups.

One possible answer to the question posed in the Introduction, i.e., how vocal students can prevent MPA symptoms from impairing their performances, may be found by a reduction in experientially avoidant behavior. Once students identified the particular ways they engaged in experientially avoidant behavior, they were encouraged to let go of attempts to control MPA symptoms and continually re-direct attention onto achieving valued outcomes in their performances, i.e., expressing emotion, while *simultaneously* experiencing MPA. In so doing, it is possible they became less impaired by their MPA symptoms because they learned to respond to them more flexibly. This possibility is strengthened by the significant improvements on the ACQ at post-treatment and both follow-ups (see ACQ scores in Table 2). Higher scores on the ACQ indicate a higher confidence in one's ability to tolerate anxiety. In fact, the idea that MPA symptoms impair performance may be believed more strongly by vocalists with higher levels of cognitive fusion. According to ACT, fusion is maintained when our thoughts have literal dominance over our behavioral responses and we become increasingly rigid in our efforts to avoid uncomfortable thoughts or emotions (Hayes et al., 2011). If believed literally, a thought like “My anxiety impairs my singing ability” may grab hold of one's behavior in this sense, as long as they are unwilling to experience it. However, if not taken literally, one can choose to persist with performing even with it present. Thus, while MPA symptoms certainly *interfere* with vocalists' physical capabilities, perhaps they are only impairing if one is unwilling to perform with them present.

Although symptom reduction was not a goal, students' MPA symptoms were significantly reduced at post-treatment and both follow-ups, a result shared by the violinist (Juncos and Markman, 2015). Students began treatment in a clear state of dysfunction, as indicated by significantly elevated KMPAI scores, however, their KMPAI scores at post-treatment and both follow-ups were significantly lower than scores at both baselines (Table 2). Moreover, their KMPAI scores fell below the clinical cutoff at during the follow-up period (Table 2), a result also shared by the violinist (Juncos and Markman, 2015).

The students also appeared to improve their average performance quality at post-treatment. This finding is important for two reasons. First, the significant improvements in Judges 1 and 3's ratings are consistent with findings from existing clinical and sport research involving ACT. These studies show ACT and related therapies can significantly improve performance on observer-rated behavioral tasks after treatment, i.e., a social skills task for patients with social anxiety (Herbert et al., submitted), a public speaking task for patients with public speaking anxiety (Block, 2002; Glassman et al., 2016), and a stressful task for patients with specific phobia (Wagener and Zettle, 2011). Also, the use of an ACT-related therapy with athletes, i.e., the Mindfulness-Acceptance-Commitment program, or “MAC” program (Gardner and Moore, 2007), has been shown to improve student and professional athletes' performance skills for 10+ years, some of whom to a significant degree (Gardner and Moore, 2004, 2007; Wolanin, 2005; Lutkenhouse, 2007; Schwanhausser, 2009; Hasker, 2010; Plemmons, 2015; Lutkenhouse et al., unpublished manuscript). In fact, the MAC is now considered “possibly efficacious” as a performance enhancement program for athletes and may soon reach the well-established level once another successful RCT is published (Gardner and Moore, 2012). Athletes in MAC studies rate the approach as credible and acceptable, therefore, musicians may also find a therapy like ACT to be helpful in improving performance quality, possibly more than CBT.

Secondly, the results from the judges' MPQ ratings are interesting, because they suggest a potential cultural difference in how the judges approached rating the performances. Judges 1 and 3 are both classically trained musicians, whereas Judge 2 is a professional performance coach for rock/pop musicians. It is possible Judge 2 was more forgiving of students' mistakes than Judges 1 or 3, because he graded performances in which students made noticeable mistakes more highly than Judges 1 or 3. Three students made noticeable mistakes during their pre-treatment performances; they were noticeable because each student stopped performing momentarily and resumed after apologizing to the accompanist. No noticeable mistakes were observed during the post-treatment performances, however. When asked to explain his approach, Judge 2 said he was more concerned with how well those students recovered after making mistakes; if they were able to finish strong in spite of the mistake, he would still grade them highly. Judges 1 and 3 did not appear to take the same approach. They rated those same performances lower than Judge 2. This makes sense considering the cultural context. Classical musicians are trained to perform in a very technically skilled way, whereas rock/pop musicians are not often held to the same rigorous standard (some of them may have no formal music training at all). Making a mistake may be perceived as more detrimental within classical music culture than in rock/pop culture. Therefore, the improvement in average performance quality that Judges 1 and 3's ratings revealed is even more significant considering it was noticed by classically trained musicians, a somewhat harder “audience” to impress.

Lastly, students were able to significantly reduce shame over having MPA by post-treatment. This result was not directly attributed to the ACT treatment, but rather to students' committing to perform regularly in front of one another. By performing in front of their peers who also had MPA, it was likely students were able to form helpful bonds with one another over having MPA. Considering how many university students are affected by MPA (~20% of students), such bonding opportunities may be essential in helping to normalize MPA as part of the common experience it apparently is for so many university students. ACT would lend itself well to this normalization, because it encourages clients to commit to regularly engaging in valued behavior, despite the presence of unwanted symptoms.

## Limitations and future directions

Given this study had a small sample and no control group, generalizations about the effectiveness of ACT as an MPA treatment are not possible. To compensate for this, both within-group comparisons and criteria typically used for determining clinically significant changes in single-case designs were made when evaluating the significance of changes on self-report measures. Self-report data is also limited, because it relies on participants' subjective ratings of themselves and inevitably leads to some degree of variability in scores, as it did here. All of the assessment measures used herein had acceptable validity and reliability levels, though some measures/subscales of measures did not have good enough internal consistency levels and should be replaced with more robust measures in future studies (VLQ's Consistency subscale α = 0.60, PHLMS' Awareness subscale α = 0.75, ESS α = 0.72). The VLQ may also be inappropriate for undergraduate and graduate students, because some of its items do not typically apply to them, i.e., questions about parenting.

A future ACT study involving music performance quality ratings should make use of a more appropriate rating scale. Given the apparent lack of psychometric data for the MPQ, it is possible that it lacks good validity and reliability levels. The MPQ does not operationalize its five domains (pitch production, rhythm/tempo, technical skill, expressiveness, tone), so the raters in this study may have understood its items differently. This could explain why the agreement was so low between raters 1 and 3, ICC (3, 2) = 0.31. Considering how common it is for expert raters to disagree when rating music performance quality (Wapnick et al., 2005; Wesolowski et al., 2015), a future study should certainly use a scale with published psychometric data.

Future studies should also include larger samples, random assignment into a control or treatment group, and a second treatment condition to compare ACT's effectiveness with that of another well-established therapy, i.e., CBT with exposure. Lastly, participants with more severe MPA and co-morbid depression should also be included, in order to compare outcomes between ACT and Psycho-dynamically oriented therapy, a treatment that has already shown promise in treating more severe cases (Kenny et al., 2014, 2016; Kenny and Holmes, 2015; Kenny, 2016). Like CBT, ACT may be more effective in treating mild to moderate cases only.

## Conclusion

This study was the first to apply ACT to the treatment of MPA with university vocalists as participants. It also offered a possible solution to a concern shared by many student vocalists, how to prevent physical MPA symptoms from impairing performance, by encouraging them to reduce experientially avoidant behaviors and re-direct attention onto achieving valued performance outcomes. The results were promising and suggest ACT is an exciting new treatment option for MPA that should be further researched. ACT is practical because it does not require costly equipment or a certification to be administered, so it could benefit a larger number of musicians with MPA. It also allows for creativity on part of the therapist in developing values-based techniques for clients to use. For this reason ACT may appeal more to musicians who feel unsure if a therapist can help them, because their own values help personalize the therapy for them, making it more relevant and fun. For more information on ACT please visit the Association for Contextual Behavioral Science's webpage, at www.contextualscience.org.

## Ethics statement

This study was carried out in accordance with the recommendations of Rider University Institutional Review Board's Human Subjects Research Guidelines with written informed consent from all subjects. All subjects gave written informed consent in accordance with the Declaration of Helsinki. The protocol was approved by the Rider University Institutional Review Board. The consent procedure involved a thorough explanation of why the study was being conducted (to investigate ACT as a treatment for MPA with student vocalists as participants), why the participants were selected (because their MPA was debilitating to them), inclusion and exclusion criteria, how long the study would take place, what the students would be asked to do as participants, possible benefits and risks associated with this treatment, alternative treatments available for MPA if interested participants did not consent, a reminder that participation is voluntary and may be terminated at any time, and a thorough explanation of confidentiality and security procedures for protected health information. Any interested students who suffered from a co-morbid disorder in addition to the MPA, i.e., major depression, substance abuse, eating disorder, etc., were not included in the study and were referred for counseling instead.

## Author contributions

DJ, GH, and KD designed the study and obtained IRB approval; DJ and EK recruited and evaluated subjects; DJ provided the full 12-session treatment to all subjects and wrote the study's manuscript, and all authors edited the manuscript and provided their final approval for it. Also, GH provided clinical supervision and statistical consultation during and after the study; KD gained faculty endorsement to allow the study to take place; SG provided accompaniment during all performances and helped design the performance schedule; PT and KD provided performance quality ratings; MM and JS provided therapist adherence ratings, and EK helped set up all performances.

### Conflict of interest statement

The authors declare that the research was conducted in the absence of any commercial or financial relationships that could be construed as a potential conflict of interest.
